# Ventrolateral ventromedial hypothalamic nucleus GABA neuron adaptation to recurring Hypoglycemia correlates with up-regulated 5′-AMP-activated protein kinase activity

**DOI:** 10.3934/Neuroscience.2021027

**Published:** 2021-09-03

**Authors:** Abdulrahman Alhamyani, Prabhat R Napit, Haider Ali, Mostafa MH Ibrahim, Karen P Briski

**Affiliations:** School of Basic Pharmaceutical and Toxicological Sciences, College of Pharmacy, University of Louisiana Monroe, Monroe, LA 71201, USA

**Keywords:** recurrent insulin-induced hypoglycemia, ventrolateral ventromedial hypothalamic nucleus-ventrolateral part, glutamate decarboxylase, AMPK, adrenergic receptor

## Abstract

Gamma-aminobutyric acid (GABA) acts on ventromedial hypothalamic targets to suppress counter-regulatory hormone release, thereby lowering blood glucose. Maladaptive up-regulation of GABA signaling is implicated in impaired counter-regulatory outflow during recurring insulin-induced hypoglycemia (RIIH). Ventromedial hypothalamic nucleus (VMN) GABAergic neurons express the sensitive energy gauge 5′-AMP-activated protein kinase (AMPK). Current research used high-neuroanatomical resolution single-cell microdissection tools to address the premise that GABAergic cells in the VMNvl, the primary location of ‘glucose-excited’ metabolic-sensory neurons in the VMN, exhibit attenuated sensor activation during RIIH. Data show that during acute hypoglycemia, VMNvl glutamate decarboxylase_65/67_ (GAD)-immunoreactive neurons maintain energy stability, yet a regional subset of this population exhibited decreased GAD content. GABA neurons located along the rostrocaudal length of the VMNvl acclimated to RIIH through a shift to negative energy imbalance, e.g. increased phosphoAMPK expression, alongside amplification/gain of inhibition of GAD profiles. Acquisition of negative GAD sensitivity may involve altered cellular receptivity to noradrenergic input via α_2_-AR and/or β_1_-AR. Suppression of VMNvl GABA nerve cell signaling during RIIH may differentiate this neuroanatomical population from other, possibly non-metabolic-sensory GABA neurons in the MBH. Data here also provide novel evidence that VMNvl GABA neurons are direct targets of glucocorticoid control, and show that glucocorticoid receptors may inhibit RIIH-associated GAD expression in rostral VMNvl GABAergic cells through AMPK-independent mechanisms.

## 1. Introduction

Insulin-induced hypoglycemia (IIH) is a persistent complication of strict glycemic management of type I diabetes mellitus [1],[2]. Maintenance of high energy-demand nerve cell functions requires a continuous supply of glucose, the primary metabolic fuel to the brain. Hypoglycemic neuro-glucopenia triggers hypothalamic activation of counter-regulatory outflow to increase plasma glucose. The mediobasal hypothalamus (MBH) integrates nutrient, endocrine, and neurochemical signals to regulate glucostasis. The ultra-sensitive energy gauge 5′-AMP-activated protein kinase (AMPK) monitors cellular energy instability in the MBH and acts there to control counter-regulation [3],[4]. The ventromedial hypothalamic nucleus (VMN), a prominent neuroanatomical component of the MBH, is a likely source of AMPK gluco-regulatory signaling as hypoglycemia stimulates AMPK phosphorylation in VMN neurons that express neurotransmitters that inhibit (gamma-aminobutryic acid; GABA) or enhance (nitric oxide; NO) counter-regulatory hormone secretion [5],[6].

Recurrent insulin-induced hypoglycemia (RIIH) triggers hypoglycemia-associated autonomic failure, a pathophysiological maladaptation to falling glucose levels that manifests as impaired glucose counter-regulation, and diminished hypoglycemic awareness. Antecedent hypoglycemia-associated augmentation of MBH GABA release is implicated in counter-regulatory dysfunction [7],[8]. Our previous studies showed that the GABA marker protein glutamate decarboxylase_65/67_ (GAD) is up-regulated in whole VMN tissue during RIIH [9]. As sampling methods that include tissue from all VMN substructures raise the possibility of inclusion of multiple GABA nerve cells populations, resulting averaged analytical measures may obscure changes to the function of GABAergic neurons that are relevant to glucoregulation. Hence, there is a critical need to ascertain the location of GABA nerve cells that become desensitized to RIIH. Mapping of the distribution of "glucose-excited” neurons in the VMN shows that nerve cells that decrease synaptic firing in response to acute reductions in substrate fuel supply reside primarily in the ventrolateral VMN (VMNvl) [10]. VMNvl GABAergic neurons are thus a credible source of hypoglycemia-sensitive glucose-lowering signaling. Current research used combinatory immunocytochemistry/laser-catapult microdissection/Western blot technology to procure pure GABA nerve cell samples from the VMNvl to address the premise that these cells exhibit correlated changes in GAD and pAMPK protein expression in reaction to acute hypoglycemia, and moreover, that these responses are each attenuated during RIIH. Reactivity of VMNvl GAD profiles to hypoglycemia varies along the longitudinal axis of this structure [11]; here, GABA neurons were obtained separately from rostral, middle, and caudal VMNvl segments for Western blot analyses.

The catecholamine neurotransmitter norepinephrine (NE) links hindbrain metabolic-sensory A2 noradrenergic neurons in the hindbrain caudal dorsal vagal complex with the VMN and other forebrain metabolic loci [12]. Exogenous NE injection to the VMN alters whole-tissue GAD protein expression [13],[14]. VMN GABAergic neurons are likely direct targets for noradrenergic control as these cells type express alpha_1_-adrenergic receptor (α_1_-AR), alpha_2_-adrenergic receptor (α_2_-AR), and beta_1_-adrenergic receptor (β_1_-AR) proteins [13],[15]. Reports that serial exogenous NE administration to the MBH suppresses counter-regulatory hormone secretion during ensuing hypoglycemia infer that counter-regulatory collapse may be mediated by recurring AR stimulation during RIIH [16]. A corollary aim of work carried out here was to investigate the hypothesis that VMNvl GABAergic nerve cell receptivity to NE signaling may be amplified by AR variant up-regulation during RIIH.

Hypoglycemia stimulates adrenal glucocorticoid hormone secretion, which elevates circulating glucose by stimulation of hepatic gluconeogenesis and inhibition of muscle and adipose tissue glucose uptake [17]. Classical/type II glucocorticoid receptors (GR) are expressed in several neural structures involved in glucostasis, including the VMN [18]–[20]. Using a rodent model for RIIH incorporating serial subcutaneous intermediate-acting insulin injection, we found that central GR antagonism prior to antecedent hypoglycemia prevents RIIH-associated diminution of transcriptional activation of GR-expressing hypothalamic neurons and averts reductions in counter-regulatory hormone release [21]. Current research examined the premise that VMNvl GABAergic neurons express GR, and that GR signaling during antecedent hypoglycemia is necessary for acclimating changes in GABA transmission during RIIH.

## 2. Materials and methods

### 2.1. Animals

Adult male Sprague Dawley rats, 3–4 months of age, were housed 2–3 per cage, according to sex, under a 14 hr light/10 hr dark cycle (lights on at 05.00 h), and allowed *ad-libitum* access to standard laboratory rat chow and water. These rodents have an average lifespan of 2–3 years. Animals were acclimated to daily handling prior to experimentation. All surgical and experimental protocols were conducted in accordance with the NIH Guide for Care and Use of Laboratory Animals, 8^th^ Edition, under approval by the ULM Institutional Animal Care and Use Committee.

### 2.2. Experimental design

To investigate effects of single versus serial hypoglycemia on VMNvl GABA neuron transmitter marker and AR variant protein expression, randomly-assigned groups of rats were injected subcutaneously (*sc*) with vehicle (V; sterile insulin diluent; Eli Lilly & Co., Indianapolis, IN) or neutral protamine Hagedorn insulin (I; 5.0 U/kg bw [9]) at 10.00 hr, over the four consecutive days of the study, as follows: group 1 (n = 4): V injection on days 1–4; group 2 (n = 4): V injection on days 1–3, I injection on day 4; group 3 (n = 4): I injection on days 1–4. Brains were collected at sacrifice at 11.00 hr on day 4, snap-frozen in liquid nitrogen-cooled isopentane, and stored at −80 °C. Plasma was stored at −20 °C for glucose measurement. This one hour post-injection time point coincides with the hypoglycemic nadir observed after administration of this intermediate-release insulin formulation, which does not vary between single versus serial injection of this drug, but does correlate with diminished counter-regulatory hormone secretion during RIIH [22]. Effects of antecedent GR antagonism on RIIH-associated patterns of VMNvl GABA nerve cell GAD and AR protein expression were investigated by bilateral intra-VMN delivery of vehicle (V; propylene glycol; group 1, n = 4) or the GR antagonist RU486 (mifepristone [23],[24]; 0.5 µg/0.5 µL; group 2, n = 4) at 09.45 hr, followed by insulin injection at 10.00 hr on days 1–3. Animals were implanted seven days prior with a 26-gauge double stainless-steel cannula guide (prod no. C235G-1.2/SPC; P1 Technologies, Roanoke, VA) aimed at the VMN, at the following coordinates: 2.85 mm posterior to *bregma*; 0.6 mm lateral to midline; 9.0 mm below skull surface, as described [14]. Bilateral infusion into the VMN by a double 33-gauge internal injection cannula (C235I-1.2/SPC; Plastics One) was carried out over a two minute period using a Genie Touch syringe pump (Lucca Technologies, Harwinton, CT). On day 4, animals in both groups were injected with insulin at 10.00 hr; brain tissue was collected at sacrifice at 11.00 hr on the same day.

### 2.3. Laser-catapult microdissection of VMNvl GABAergic neurons

Each brain was cut into consecutive 10 µm-thick frozen sections over the rostrocaudal length of the VMN, e.g. 1.8–3.3 mm posterior to *bregma*, and mounted on polyethylene naphthalate membrane-coated slides (Carl Zeiss Microscopy, LLC, White Plains, NY). Sections were processed by immunoperoxidase histochemistry to visualize glutamate decarboxylase_65/67_ (GAD)-immunoreactive (-ir) neurons, as described [12],[13]. Briefly, tissues were fixed with acetone, then blocked with 2% normal goat serum (NGS) in Tris-buffer saline, pH 7.4 (TBS) prior to incubation (48 hr) with rabbit primary antibodies against GAD (1:1,000; prod. no. ABN904; Millipore Sigma, Burlington, MA) in TBS containing 0.05% Triton X-100 (TBS-Tx). Sections were next exposed to horseradish peroxidase-labeled goat anti-rabbit IgG (1:1,000; prod no: PI-1000; Vector Laboratories, Burlingame, CA) in TBS-Tx-2% NGS, followed by ImmPACT 3,3′-diaminobenzidine peroxidase substrate kit reagents (product no. SK 4105; Vector Lab.). For each animal, GAD-ir neurons were collected using a Zeiss P.A.L.M. UV-A microlaser IV to create separate lysate pools for rostral (−1.8 to −2.3 mm), medial (−2.3 to −2.8 mm) and caudal (−2.8– −3.3 mm) segments of the VMNvl.

### 2.4. Western blot analysis of VMNvl GABA neuron protein expression

For each treatment group, lysate aliquots from individual subjects were combined within each rostrocaudal segment to create three separate sample pools for each protein of interest. Equivalent sample protein mass from each pool was separated in Bio-Rad Stain-Free 10% gradient acrylamide gels (prod. no. 161–0183, Bio-Rad Laboratories Inc., Hercules CA Hercules, CA). Prior to trans-blotting to 0.45 µm PVDF-Plus membranes (prod. no. 1212639; Data Support Co., Panorama City, CA), gels were UV light-activated (2 min) in a BioRad ChemiDoc TM Touch Imaging System. Membranes were blocked with TBS containing 0.1% Tween-20 and 2% bovine serum albumin prior to incubation with rabbit primary polyclonal antisera against GAD (1:1,000; prod. no. ABN904; Millipore Sigma), AMPK_α1/2_ (1:2000; prod. no. 2532S; Cell Signaling Technology, Inc., Danver, MA), phosphoAMPK-Thr 172 (pAMPK) (1:2000; prod. no. 2531S; Cell Signaling Technol.), α_1_-AR/ADRA1A (1:1000; prod. no. NB100-78585; Novus Biologicals, Littleton, CO), α_2_-AR/ADRA3A (1:1000; prod. no. NBP1-67832, Novus Biol.), or β_1_-AR/ADRB1 (1:1000; prod. no. NBP1-59007; Novus Biol.). Membranes were then incubated with a horseradish peroxidase-labeled goat anti-rabbit secondary antiserum (1:5,000; prod. no. NEF812001EA; PerkinElmer, Waltham, MA), before exposure to SuperSignal West Femto maximum-sensitivity chemiluminescent substrate (prod. no. 34096; ThermoFisherScientific, Waltham, MA). Chemiluminescence band optical density (O.D.) values for target proteins were normalized to total protein quantified in the sample lane, e.g. the lane in which that protein was electrophoresed, using Bio-Rad proprietary stain-free imaging gel technology and ChemiDoc MP instrumentation with Image Lab™ 6.0.0 software, as described at http://www.bio-rad.com/en-us/applications-technologies/stain-free-imagingtechnology?ID=NZ0G1815. This superior method for Western blot normalization distinctly diminishes data variability through improved measurement accuracy and precision [25],[26]. Precision plus protein molecular weight dual color standards (prod. no. 161–0374, Bio-Rad) were included in each Western blot analysis.

### 2.5. Statistics

Mean normalized protein O.D. values for GABA neurons within rostral, middle, and caudal VMNvl segments were evaluated by one-way analysis of variance and Student-Newman-Keuls *post-hoc* test or Student's *t* test. Differences of p < 0.05 were considered significant. In each figure, statistical differences between specific pairs of treatment groups are denoted as follows: *p < 0.05; **p < 0.01; ***p < 0.001.

## 3. Results

**Figure 1.**
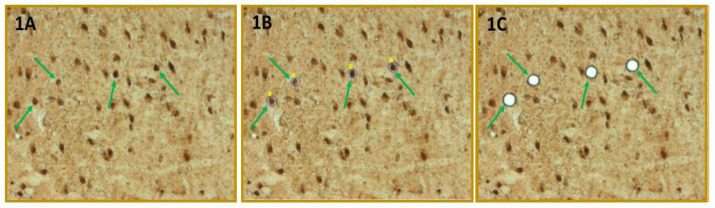
Laser-Catapult Microdissection of Immunolabeled Ventrolateral Ventromedial Hypothalamic Nucleus (VMNvl) Gamma-Aminobutyric Acid (GABA) Neurons. VMN cells were identified *in situ* for glutamate decarboxylase_65/67_ (GAD)-immunoreactivity (-ir). Representative GAD-ir-labeled neurons are indicated by blue arrows. Areas depicted in Figure 1A were re-photographed after positioning of a continuous laser track (depicted in blue) around individual GAD-ir neurons (Figure 1B) and subsequent ejection of those cells by laser pulse (Figure 1C). Note that this microdissection technique causes negligible destruction of surrounding tissue and minimal inclusion of adjacent tissue.

Figure 1 depicts selective laser-catapult microdissection of VMNvl GABAergic neurons. GAD-immunoreactive (ir) nerve cells were identified *in situ* by immunoperoxidase staining (Figure 1A). Green arrows indicate representative immunolabeled neurons. Figures 1B and 1C show sequential actions, i.e. computer-assisted positioning of a continuous laser cut (shown in blue) surrounding individual GAD-ir neurons, followed by application of a laser pulse to remove each circumdissected cell from the plain of the tissue section, that result in nerve cell harvesting without destruction of surrounding tissue.

**Figure 2.**
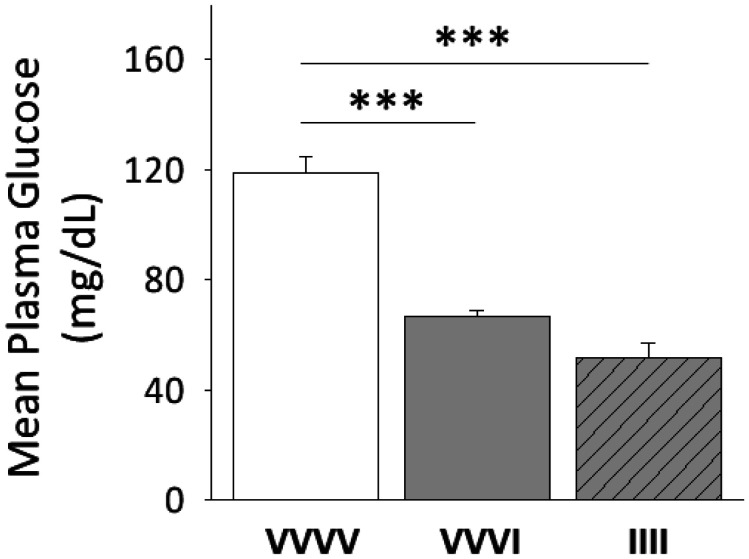
Plasma Glucose Measures One Hour after One versus Four Injections, once per day, of Neutral Protamine Hagedorn Insulin (I). Data depict mean glucose measures + S.E.M. for VVVV (n = 4 rats), VVVI (n-4 rats), and IIII (n = 4 rats) treatment groups.

Data in Figure 2 show that plasma glucose concentrations were significantly decreased [F_(2,9)_ = 41.4; *p* < 0.0001] to an equivalent extent at +1 hr after single [VVVI; solid gray bars] or serial [IIII; diagonal-striped gray bars] *sc* I dosing compared to euglycemic controls [VVVV; solid white bars].

**Figure 3.**
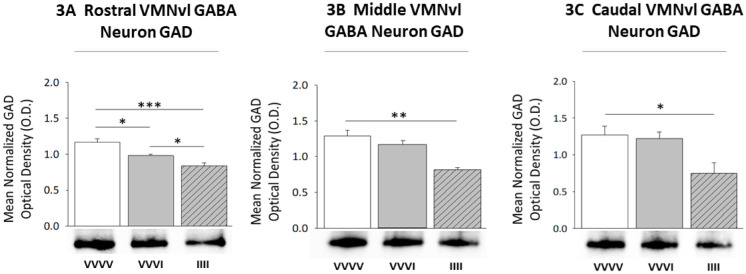
Effects of Single versus Serial Exposure to Insulin-Induced Hypoglycemia on Ventrolateral Ventromedial Hypothalamic Nucleus (VMNvl) Gamma-Aminobutyric Acid (GABA)-ergic Neuron Glutamate Decarboxylase (GAD) Protein Expression. GAD-immunoreactive neurons were harvested by laser-catapult microdissection from rostral, middle, and caudal segments of the VMNvl from rats injected with vehicle (V) alone over the four day experiment (VVVV; group 1; n = 4 rats) or neutral protamine Hagedorn insulin (I; 5.0 U/kg bw) either once on day 4 (VVVI; group 2; n = 4 rats) or on study days 1–4 (IIII; group 3; n = 4 rats). Data depict mean normalized rostral (Figure 3A), middle (Figure 3B), and caudal (Figure 3C) VMNvl GABAergic nerve cell GAD protein optical density (O.D.) measures + S.E.M. for VVVV, VVVI, and IIII treatment groups. *p < 0.05; **p < 0.01; ***p < 0.001.

Figure 3 depicts effects of single versus serial exposure to hypoglycemia on rostral (Figure 3A; F_(2,9)_ = 12.61; *p* = 0.0005), middle (Figure 3B; F_(2,9)_ = 41.4; *p* < 0.0001), and caudal (Figure 3C; F_(2,9)_ = 6.77; *p* = 0.014) VMNvl GABAergic neuron GAD protein expression. One episode of hypoglycemia caused significant down-regulation of GAD in GABA neurons in the rostral, but not other segments of the VMNvl, compared to euglycemic controls [VVVI (solid gray bars) versus VVVV (solid white bars)]. GABA neurons in each VMNvl region exhibited diminution of GAD profiles during the fourth of four daily episodes of hypoglycemia versus controls [IIII (diagonal-striped gray bars) versus VVVV (solid white bars)]. In rostral VMNvl GABA nerve cells, GAD protein profiles were reduced to a significantly greater extent after the fourth versus first insulin injection [IIII (diagonal-striped gray bar) versus VVVI (solid gray bar)].

**Figure 4.**
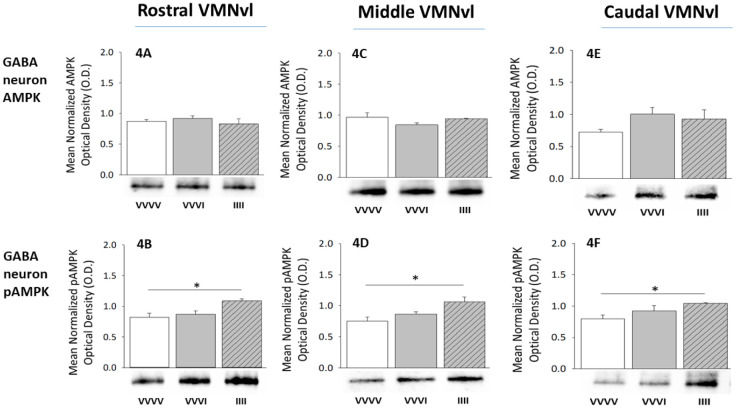
Patterns of 5′-AMP-Activated Protein Kinase (AMPKJ) and PhosphoAMPK (pAMPK) Expression in VMNvl GABAergic Neurons during Acute versus Recurring Hypoglycemia. Data show mean normalized GABA nerve cell AMPK [rostral (Figure 4A, F(2,9) = 14.91; p = 0.0012), middle (Figure 4C, F(2,9) = 7.30; p = 0.011), caudal (Figure 4E, F(2,9) = 6.20; p = 0.0087) VMNvl segment] and pAMPK [rostral (Figure 4B, F(2,9) = 5.92; p = 0.01), middle (Figure 4D, F(2,9) = 14.32; p = 0.0014), caudal (Figure 4F, F(2,9) = 13.17; p = 0.0018) VMNvl segment] O.D. measures + S.E.M. for VVVV (n = 4 rats), VVVI (n = 4 rats), and IIII (n = 4 rats) treatment groups. *p < 0.05.

Effects of one versus four insulin dosages on VMNvl GABAergic nerve cell AMPK and pAMPK protein content, according to rostrocaudal segment, are shown in Figure 4. At each level, GABA neuron AMPK levels were unaffected by either single or serial exposure to hypoglycemia (Figure 4A, rostral VMNvl; Figure 4C, middle VMNvl; Figure 4E, caudal VMNvl). Data presented in Figures 4B, 4D, and 4E show that nerve cell pAMPK profiles were refractory to acute hypoglycemia, but were significantly up-regulated during RIIH throughout the rostrocaudal length of the VMNvl.

**Figure 5.**
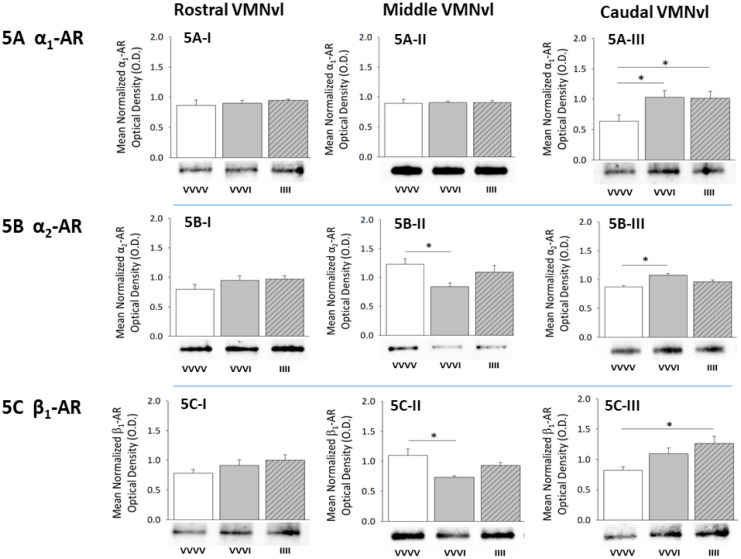
Impact of Single versus Serial Insulin Dosing on VMNvl GABAergic Neuron Adrenergic Receptor (AR) Variant Protein Expression. Data depict mean normalized GABA nerve cell α_1_-AR (Figure 5A), α_2_-AR (Figure 5B), and β_1_-AR (Figure 5C) protein O.D. values + S.E.M. for subsets of neurons collected from the rostral [left-hand column], middle [middle column], and caudal [right-hand column] VMNvl of animals injected with V only (VVVV; n = 4), a single insulin dose (VVVI; n = 4), or four insulin dosages one dosage per day (IIII; n = 4). *p < 0.05.

Data presented in Figure 5 illustrate α_1_-AR (Figure 5A), α_2_-AR (Figure 5B), and β_1_-AR (Figure 5C) protein expression patterns in VMNvl GABA neurons in response to acute or recurring hypoglycemia. GABAergic neurons collected from the rostral VMNv exhibited no change in any AR; variant after single or serial insulin injection (Figures 5A-I, 5B-I, and 5C-I). Middle VMNvl GABA nerve cell exhibited down-regulated α_2_-AR (Figure 5B-II; F_(2,9)_ = 6.44; *p* = 0.016) and β_1_-AR (Figure 5C-II; F_(2,9)_ = 6.08; *p* = 0.018) expression during acute hypoglycemia, but both protein profiles were refractory to RIIH. Exposure to a single bout of hypoglycemia elevated α_1_-AR (Figure 5A-III, F_(2,9)_ = 6.76; *p* = 0.0064) and α_2_-AR (Figure 5B-III, F_(2,9)_ = 7.30; *p* = 0.0041) levels in caudal VMNvl GABAergic neurons. In this regional cell subset, the former protein response persisted during RIIH, whereas the latter response was lost as a consequence of antecedent hypoglycemia. These neurons also exhibited up-regulation of β_1_-AR during chronic, but not acute hypoglycemia (Figure 5C-III; F_(2,9)_ = 6.05; *p* = 0.0095).

**Figure 6.**
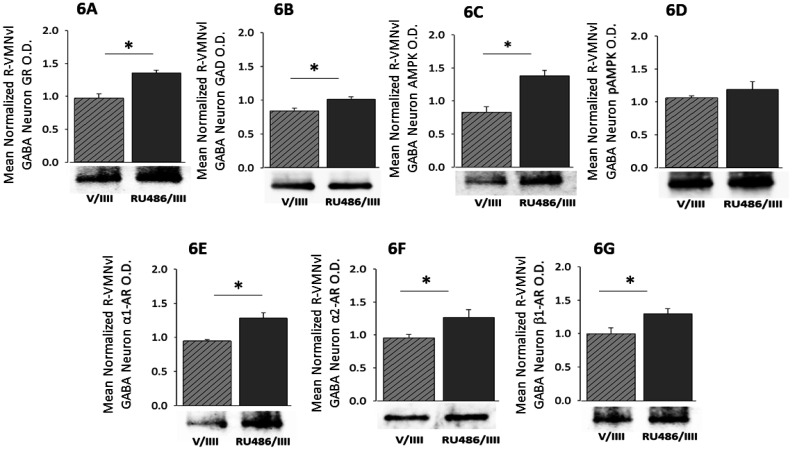
Role of Antecedent Glucocorticoid Receptor (GR) Signaling on RIIH-Associated Patterns of GAD and AR Protein Expression in Rostral VMNvl GABAergic Neurons. Data show mean normalized Rats were pretreated by intra-VMN administration of vehicle (group 1; n = 4 rats) or the GR antagonist RU486 (group 2; n = 4 rats) prior to insulin injection on study days 1–3. Animals received a fourth and final insulin dose on day 4. Data show mean normalized GR (Figure 6A), GAD (Figure 6B), AMPK (Figure 6C), pAMPK (Figure 6D), α_1_-AR (Figure 6E), α_2_-AR (Figure 6F), or β_1_-AR (Figure 6G) protein O.D. measures + S.E.M. for GABA nerve cells collected from V/IIII and RU486/IIII treatment groups. *p < 0.05.

Figure 6 illustrates effects of antecedent hypoglycemia-associated VMN GR signaling on rostral VMNvl GABAergic nerve cell AMPK activation alongside transmitter marker and AR variant protein expression during RIIH. Western blot analysis showed that these GABA neurons express GR (Figure 6A; t(5) = 5.12, p = 0.0069). Administration of the GR antagonist RU486 prior to hypoglycemia on study days 1–3 caused significant augmentation of GAD profiles during hypoglycemia on day 4 (Figure 6B) [RU/IIII (dark gray bar) versus V/IIII (diagonal-striped light gray bar); t(5) = 3.13, p = 0.02]. This pretreatment enhanced total AMPK expression in these GABA neurons (Figure 5C; t(5) = 4.49, p = 0.01), but did not alter pAMPK content (Figure 6D; t(5) = 2.51, p = 0.22). Blockade of GR signaling during antecedent hypoglycemia increased α_1_-AR (Figure 6E), α_2_-AR (Figure 6F), and β_1_-AR (Figure 6G) protein expression in rostral VMNvl GABA neurons during RIIH. Plasma glucose concentrations did not differ between V/IIII [51.5 ± 5,6 mg/dL] versus RU/IIII [52.3 ± 4.87 mg/dL] groups at +1 hr after administration of the final I dosage.

**Figure 7.**
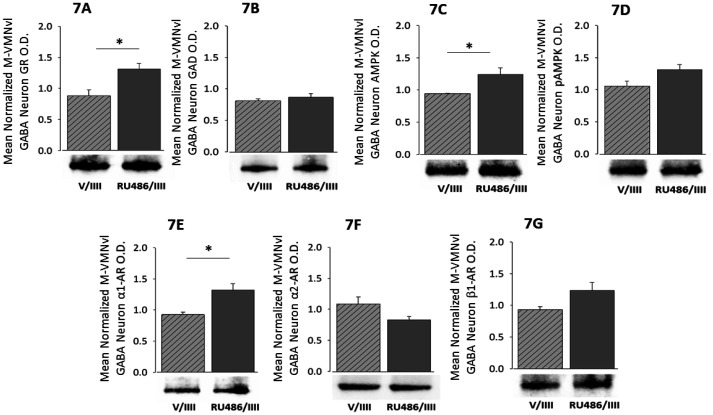
RIIH-Associated Patterns of GAD and AR Protein Expression in Middle VMNvl GABAergic Neurons; Role of Antecedent GR Signaling. Data show mean normalized GR (Figure 7A), GAD (Figure 7B), AMPK (Figure 7C), pAMPK (Figure 7D), α_1_-AR (Figure 7E), α_2_-AR (Figure 7F), or β_1_-AR (Figure 7G) protein O.D. measures + S.E.M. for GABAergic neurons collected from V/IIII (n = 4 rats) and RU486/IIII (n = 4 rats) treatment groups. *p < 0.05; **p < 0.01; ***p < 0.001; ****p < 0.0001.

Effects of antecedent GR blockade on RIIH-associated patterns of GAD and AR variant protein expression in middle VMNvl GABAergic neurons are shown in Figure 7. RU486 pretreatment up-regulated GR profiles in these cells (Figure 7A; t(5) = 3.37, p = 0.019), but did not alter GAD content (Figure 7B). GABA nerve cell AMPK (Figure 7C; t(5) = 2.96, p = 0.031), but not pAMPK (Figure 7D) expression was up-regulated during RIIH by GR antagonism. Blockade of GR signaling on days 1–3 augmented α_1_-AR (Figure 7E; t(5) = 3.64, p = 0.023), but not α_2_-AR (Figure 7F) or β_1_-AR (Figure 7G) protein expression in middle VMNvl GABA neurons during RIIH.

**Figure 8.**
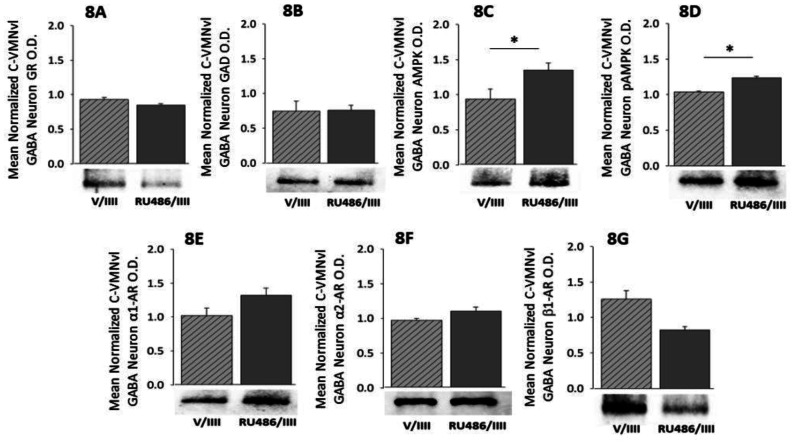
Effects of GR Antagonism on RIIH-Associated Patterns of GAD and AR Protein Expression in Caudal VMNvl GABAergic Neurons. Data show mean normalized GR (Figure 8A), GAD (Figure 8B), AMPK (Figure 8C), pAMPK (Figure 8D), α_1_-AR (Figure 8E), α_2_-AR (Figure 8F), or β_1_-AR (Figure 8G) protein O.D. measures ± S.E.M. for GABAergic neurons collected from V/IIII (n = 4) and RU486/IIII (n = 4) treatment groups. *p < 0.05; **p < 0.01; ***p < 0.001; ****p < 0.0001.

As shown in Figure 8, GR antagonism had no effect on caudal VMNvl GABAergic GAD expression (Figure 8B), but augmented patterns of AMPK (Figure 8C; t(5) = 2.39, p = 0.034) and pAMPK (Figure 8D; t(5) = 6.81, p = 0.002) expression in these cells. RU486 pretreatment did not modify RIIH-associated AR variant expression profiles in this nerve cell subset (Figures 8E, 8F, and 8G).

## 4. Discussion and conclusions

De-sensitization of MBH GABAergic transmission to RIIH reportedly contributes to maladaptive impairment of counter-regulatory hormone secretion [7],[8]. Current research utilized a high-resolution dissection approach for neurotransmitter cell type sampling to address the premise that GABAergic neurons in the VMNvl, the primary source of glucose-lowering metabolic-sensory signaling in the VMN, become refractory to hypoglycemic energy imbalance. Outcomes provide unsuspected evidence that RIIH-associated suppression of VMNvl GABAergic signaling over the rostrocaudal length of the VMNvl may offset acclimated normalization of transmission by other, likely non-metabolic-sensory GABA neurons in the MBH.

Evidence here that VMNvl GABA neurons express the metabolic-sensory biomarker AMPK suggests that their electrophysiological reactivity to neuro-gluocopenia may be similar to that described for VMNvl “glucose-excited” neurons [10]. As GABA neurons investigated here showed no change in pAMPK content during acute hypoglycemia, reductions in GAD profiles in cells taken from the rostral VMNvl imply that hypoglycemia-associated diminution of neurotransmitter signaling by this cell subset is mediated by cues other than cellular energy status. Refractoriness of GAD protein in GABA neurons collected from other VMNvl segments confirms prior observations in our laboratory involving micropunch VMNvl tissue dissection [11]. Here, regional subsets of VMNvl GABAergic nerve cells exhibited divergent adjustments in AR variant protein expression during acute hypoglycemia. The lack of change in α_1_-AR, α_2_-AR, or β_1_-AR protein levels in rostral VMNvl GABA neurons suggest that hypoglycemic down-regulation of GAD expression in these cells may be NE-independent. Yet, stabilization of GABAergic signaling by middle and caudal VMNvl GABA cell groups may reflect, in part, altered sensitivity to noradrenergic input. The factors that govern VMNvl GABAergic neuron receptivity to NE during acute neuro-glucopenia remain to be characterized.

Data show that RIIH increased pAMPK expression in GABA nerve cells located throughout the rostrocaudal length of the VMNvl, findings that infer that mechanisms of adaptation to this metabolic stress may result in a negative shift in energy balance across all regional subsets. This interpretation remains speculative as analytical methods of requisite sensitivity for quantification of ATP in small-volume brain cell samples such as those acquired here do not currently exist. Thus, the prospect that augmented AMPK activity may reflect, in part, acclimated patterns of afferent neurochemical and/or hormonal input to these neurons during RIIH cannot be discounted. Concurrent adjustments in pAMPK and GAD protein profiles support the possibility of a causal link between these inverse responses. Interestingly, RIIH-associated patterns of GAD expression reflected amplification of acute negative reactivity in rostral VMNvl GABA neurons, but gain of inhibitory responsiveness in cells located in other VMNvl segments. Observations here that acute and recurring hypoglycemia impose dissimilar regulatory effects on AR variant protein expression in middle and caudal VMNvl GABA nerve cells support the view that their acquired sensitivity to RIIH may be mediated, in part, by NE.

Current results provide unique proof of GR expression by VMNvl GABAergic neurons. Effects of antecedent GR signaling on RIIH-associated patterns of GABA transmission vary, as receptor antagonism on days 1–3 of insulin dosing elevated GAD expression in neurons in rostral, but not middle or caudal segments of the VMNvl during fourth and final induction of hypoglycemia. The lack of RU486 pretreatment effects on RIIH-associated pAMPK profiles in rostral VMNvl GABA neurons infer that acclimation to prior GR stimulation may suppress GAD levels during RIIH regardless of AMPK activity state during final exposure to hypoglycemia. Alternatively, the extent to which AMPK can be activated during RIIH may be limited due to maximal phosphorylation capacity, such that RU486-initiated signals for intensification of pAMPK expression are not capable of increasing this protein profile. As prior work showed that global forebrain GR antagonism prevented habituation of hypothalamic nerve cell transactivation [27], outcomes here infer that broad GR inhibition across diverse brain loci likely masks divergent site-specific GR control of gluco-regulatory transmission. Observations here of GR-mediated amplification of GABAergic inhibitory signaling by a neuroanatomically-defined VMNvl GABA cell subset reveal that these GR elicit an outcome, e.g. augmented repression of metabolic transmission that inhibits counter-regulation, that deviates from the average effect of forebrain GR, namely attenuation of counter-regulatory outflow. Middle and caudal VMNvl GABA neurons express GR, but RIIH-associated GAD profiles in these cell subsets were refractory to control by this receptor. Possible explanations include offsetting stimulatory effects of other regulatory inputs to these neurons, or, alternatively, acclimating down-regulation of post-receptor signaling as a consequence of antecedent hypoglycemia exposure. As noted above, rostral VMNvl GABA nerve cell α_1_-AR, α_2_-AR, and β_1_-AR protein profiles were unaffected by RIIH, yet antecedent GR antagonism elevated expression of each variant after administration of a fourth and final insulin dosage. These findings suggest that inhibitory GR effects on these proteins are likely counter-balanced by stimulatory cues.

**Figure 9.**
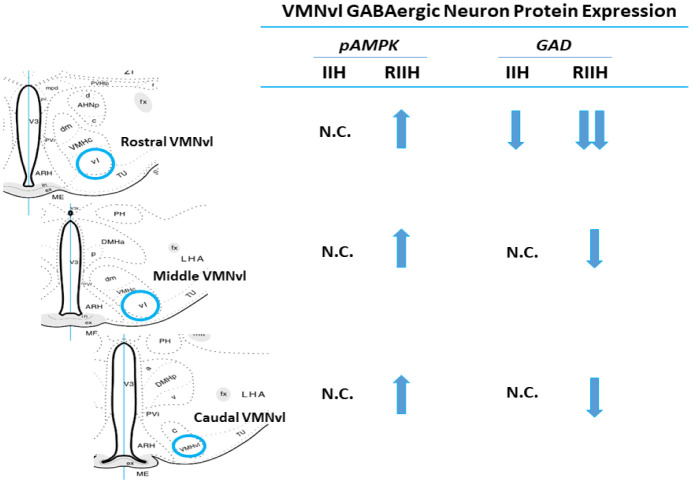
Effects of IIH versus RIIH on Rostral, Middle, or Caudal VMNvl GABAergic Neuron pAMPK and GAD protein expression. Arrows depict up- (↑) or down (↓)-regulation of mean nerve cell pAMPK or GAD levels of laser-microdissected VMNvl GAD-immunopositive neurons during acute versus recurring exposure to insulin-induced hypoglycemia. The abbreviation N.C. indicates no net change in protein content.

Results refute the likelihood that VMNvl GABA neurons participate in RIIH-associated counter-regulatory collapse, as transmitter signaling by these cells evidently declines relative to acute hypoglycemia. Correlation of exacerbation (rostral VMNvl) or gain (middle and caudal VMNvl) of GAD inhibitory response to RIIH with up-regulated pAMPK expression infers that these GABA neurons relinquish the capacity to maintain a stable energy state as a result of re-exposure to hypoglycemia (Figure 9). Thus, ill-restrained GABAergic gluco-inhibitory transmission evidently occurs elsewhere in the MBH during RIIH; further studies are need to identify that/those site(s). It would also be insightful to learn if GABA neurons that show less negative responsiveness to RIIH are metabolic-sensory in function, or if such acclimation is independent of intrinsic energy state. It is presumed that the brain gluco-regulatory network ultimately integrates information from neuroanatomically distinct GABA neuron populations that exhibit divergent acclimation of gluco-inhibitory transmission, which likely reflect adaptation to different cues and inputs, to control counter-regulatory outflow during RIIH.

It should be noted that the current study utilized non-diabetic rats rather than animals with controlled or uncontrolled diabetes. Thus, the possibility cannot be overlooked that rostrocaudal region-specific VMNvl GABAergic neuron responses to acute or repetitive hypoglycemia may differ between hyperglycemia-naïve rats versus subjects exposed to continuous or intermittent hyperglycemia. The laser-microdissection technology used here is efficacious for sampling of nerve cell bodies, but is not sufficiently refined for acquisition of axon terminals containing target neurotransmitters or biosynthetic enzymes. As a result, the question of whether effects of acute and/or repeated hypoglycemia on GAD expression profiles in projections from VMNvl GABA neurons remains unanswered at present.

In summary, research outcomes emphasize the efficacy of high-neuroanatomical resolution methodology for neurotransmitter cell type acquisition for discovery of functionally-diverse subgroups of hypothalamic gluco-regulatory neurons. Data show that during acute hypoglycemia, VMNvl GABAergic neurons maintain energy stability, yet a regional subset of this population exhibits decreased neurotransmission. Habituation of these neurons to RIIH involves a shift to negative energy imbalance alongside amplification or gain of inhibitory reactivity to this metabolic stress. This acquisition of negative sensitivity may involve altered receptivity to noradrenergic input via α_2_-AR and/or β_1_-AR. VMNvl GABAergic nerve cell energy deficit-driven signaling during RIIH may functionally distinguish this population from other, possibly non-metabolic-sensory GABA neurons in the MBH. The molecular mechanisms that determine reactivity versus non-reactivity of GABA nerve cell subsets to GR signaling during RIIH remain to be clarified.
